# Behaviour of *K. pneumoniae*|Carbapenems in Several European Countries According to Clustering Time-Series Health Data with Generalised Affinity Coefficient

**DOI:** 10.3390/antibiotics15090895

**Published:** 2026-09-11

**Authors:** Ana Paula Nascimento, Mónica Vieira, Cristina Prudêncio, Helena Bacelar-Nicolau

**Affiliations:** 1 RISE-Health, Center for Translational Health and Medical Biotechnology (TBIO), Escola Superior de Saúde (E2S), Universidade Técnica do Porto (UTP), 4200-072 Porto, Portugal; ananascimento@ess.ipp.pt (A.P.N.); mav@ess.ipp.pt (M.V.); 2 Biomatemática, Bioestatística e Bioinformática, Escola Superior de Saúde (E2S), Universidade Técnica do Porto (UTP), 4200-072 Porto, Portugal; 3 Ciências Químicas e das Biomoléculas, Escola Superior de Saúde (E2S), Universidade Técnica do Porto (UTP), 4200-072 Porto, Portugal; 4 Faculdade de Psicologia, Universidade de Lisboa (FPUL), 1649-013 Lisboa, Portugal; hbacelar@psicologia.ulisboa.pt; 5 Institute of Environmental Health, Faculdade de Medicina, Universidade de Lisboa (ISAMB-FMUL), 1649-028 Lisboa, Portugal

**Keywords:** antimicrobial resistance, *K. pneumoniae*, hierarchical cluster analysis, generalised affinity coefficient, ARIMA model

## Abstract

**Background/Objectives**: Antimicrobial resistance (AMR) constitutes a major public health and economic burden in contemporary society. This work aims to identify the patterns and temporal trends of *Klebsiella pneumoniae* resistance to carbapenems across several European countries. **Methods**: Data on *K. pneumoniae* resistance to carbapenems in several countries from 2005 to 2021 (with data available up to 2023) were retrieved from the public ECDC website in 2023. Agglomerative Hierarchical Cluster Analysis (HCA), based on the new generalised affinity coefficient, was applied to the estimated ARIMA models. **Results**: The dendrogram at the cut-off presented four clusters: the first cluster with Slovenia, Luxembourg, Italy and Estonia; the second cluster with Romania, Malta, Greece, Portugal and Spain; the third cluster Denmark, France and Czechia; and finally the fourth cluster with Lithuania, Cyprus, Finland, Austria, Belgium and Slovakia. Although the resistance values showed an increasing trend over the years in clusters II and III, the increase was more pronounced in cluster II. **Conclusions**: The application of data analysis, for the first time, using HCA based on a new similarity coefficient, namely, the generalised affinity coefficient, enables the evaluation of time-series patterns in different regions and enables the identification of clusters of countries showing similar temporal patterns of antimicrobial resistance. Carbapenem resistance in *K. pneumoniae* among countries belonging to cluster II showed increasing trends throughout the study period.

## 1. Introduction

The emergence of acquired antimicrobial resistance (AMR) is posing a major challenge, often restricting therapeutic options and leading to greater patient suffering and mortality [1,2]. In humans, the increasing levels of AMR are primarily attributed to the overuse and inappropriate use of these agents by both patients and healthcare professionals [2]. Additionally, the extensive application of antimicrobials beyond human medicine represents a significant concern. Antimicrobial agents have been increasingly utilised in plant biotechnology and in the animal sector either to treat bacterial infections or to promote growth in intensive farming practices [3,4]. Moreover, antimicrobial-resistant bacteria have also been identified in certain retail meats, poultry, and various other types of food [3,5]. Antimicrobial residues were also found in wastewater [6].

The growing prevalence of drug-resistant infections has led to increased morbidity, mortality, and healthcare costs worldwide [7]. Bacterial resistance to antimicrobial agents has been increasing in Europe over the years [8], and, to overcome this challenge, it is necessary to develop new and improved diagnostic methodologies. According to the WHO, new antimicrobials or strategies are urgently needed to treat carbapenem-resistant Gram-negative bacterial infections [9]. Addressing this issue requires not only coordinated policy action but also a deep understanding of the epidemiological patterns and trends underlying AMR, particularly across European countries. This work aims to identify patterns and temporal trends in *Klebsiella pneumoniae* resistance to carbapenems in several European (EU) countries in order to contribute to a better understanding of its dynamics and public health implications, since this is a pair considered by the WHO as a critical priority [10].

Thus, the main objective of this study is to analyse the hierarchy of cluster partitions of *K. pneumoniae* based on resistance to carbapenems in several EU countries using agglomerative hierarchical cluster analysis (HCA) with a new similarity measure, namely, the generalised affinity coefficient [11,12,13,14,15,16]. This new measure enables the identification of countries showing similar temporal patterns in *K. pneumoniae* resistance to carbapenems because it measures the similarity between the Autoregressive Integrated Moving Average (ARIMA) models fitted to the data rather than between the raw observations. In fact, ARIMA models explicitly account for temporal dependence in the data, making them suitable for the analysis of time-series patterns and trends. Therefore, this measure can identify similarities between time series based on their modelled temporal dependence structures, which may capture aspects of the underlying dynamics that are not adequately represented by conventional multivariate clustering methods applied directly to the raw observations. Moreover, as similarity is evaluated based on ARIMA models fitted to the data rather than on the raw observations, this measure allows the analysis of datasets with missing values, provided that sufficient observations are available. An alternative dissimilarity measure could have been considered. Several dissimilarity and similarity measures between time series have been proposed [17,18,19]. One of the most widely used dissimilarity measures is the Euclidean distance between the autoregressive expansions of fitted ARIMA models, proposed by Piccolo [20,21]. This distance is independent of the sign of the coefficients in the autoregressive expansions of the fitted ARIMA models. The generalised affinity coefficient also measures the similarity between ARIMA models but, in contrast to the Piccolo distance, treats ARIMA models with symmetric coefficients as distinct [13].

The analysis of temporal data is essential for identifying geographic and demographic patterns, offering deeper insights into health disparities across populations. Unlike standard studies, which have often relied on spatial autocorrelation or broad socioeconomic proxies to group countries, agglomerative HCA using the new generalised affinity coefficient with ARIMA models was applied in this study [13]. Therefore, the authors aimed to model the resistance data for the *K. pneumoniae*|carbapenem pair in each country using ARIMA models in order to characterise and compare temporal trajectories, thereby shifting the analytical focus from geographic proximity to epidemiological behaviour.

By employing this distance measure, the temporal dynamics of resistance values across 18 EU countries were studied using data from 2005 to 2021. This approach allowed grouping countries based on the evolution of their health indicators over time, enabling a clear assessment of model reliability and resistance dynamics in diverse national contexts and facilitating the identification of comparable patterns in public health outcomes.

Importantly, this methodology not only facilitates the identification of countries with similar temporal resistance patterns but also requires the selection of the most appropriate time-series models, thereby providing a basis for forecasting future resistance behaviour. ARIMA models have long been established as valuable tools for modelling temporal dependence in time-series data and forecasting a wide range of data. ARIMA models have been extensively applied in the health sector to analyse and forecast a variety of outcomes, including infectious disease transmission [22], cancer incidence and mortality [23], chronic kidney disease cases [24], and chronic respiratory disease morbidity [25]. These applications demonstrate the value of ARIMA models for modelling temporal behaviour in health data. Therefore, the use of HCA based on the generalised affinity coefficient between ARIMA models enables the assessment of patterns among different regions and facilitates the identification of groups of countries with similar temporal resistance behaviours, thereby supporting a more detailed examination of their resistance dynamics, which may require further study.

The focus of this study on *Klebsiella pneumoniae* resistance to carbapenems in several EU countries is based on being a pair considered by the WHO as a critical priority [10], indicating an urgent need to identify patterns and temporal trends in its behaviour.

## 2. Results

The data of resistant *K. pneumoniae*/carbapenem agents was requested from the public website of European Centre for Disease Prevention and Control (ECDC) [26].

The data collected contained the resistant isolate percentage values of *K. pneumoni-ae*|carbapenem pair in several countries in the EU from 2005 to 2021, obtained in July 2023.

In Table 1, the mean and standard deviation of the resistance values of the *K. pneumoniae*|carbapenem pair for each country are presented.

From Table 1, it can be observed that Greece, Romania, and Italy had the highest mean resistance values. Finland had the lowest mean resistance value.

The results of the agglomerative HCA, using the generalised affinity coefficient between the ARIMA models that best fit the resistant isolate percentage values over time (see Table A1 and Table A2), for the *K. pneumoniae*|carbapenem pair in several EU countries, are presented below.

### Agglomerative Hierarchical Cluster Analysis with Generalised Affinity Coefficient Between ARIMA Models

These ARIMA models can be used to predict the behaviour of resistance in each country, allowing better coordination of intervention and prevention actions. The ARIMA models that best fit the resistant isolate percentage values data for *K. pneumoniae* |carbapenems are presented in Appendix A. The percentage of resistant *K. pneumoniae* isolates resistant to carbapenems over time was used to fit the best ARIMA models (see Appendix A), characterised by the parameters theta and phi, which enable the calculation of the generalised affinity coefficient.

**Figure 1:**
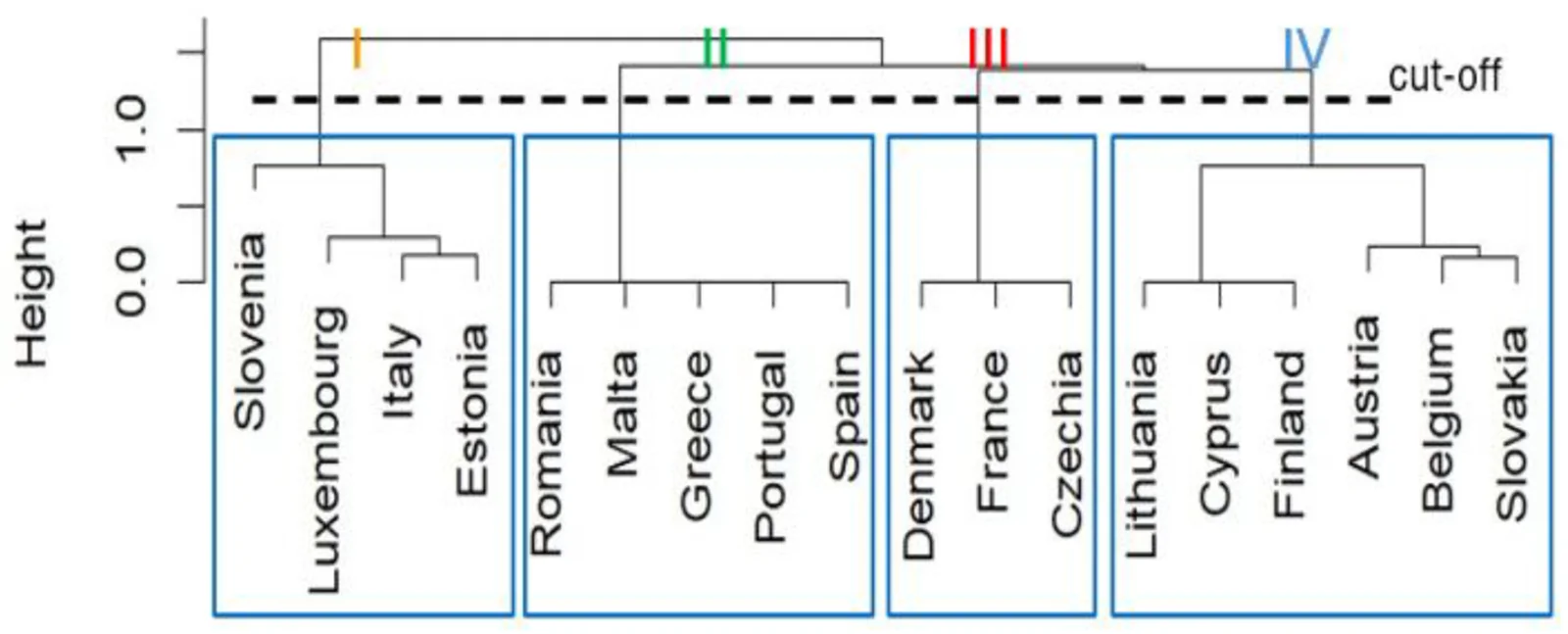
Dendrogram based on square root of 2(1-affinity) distance and average linkage aggregation criterion between countries in *K. pneumoniae*|carbapenem resistance context. I–IV represent the clusters obtained.

The dendrogram of the agglomerative HCA with average linkage aggregation criterion is shown in Figure 1.

In the dendrogram, the development of four clusters of countries can be seen, which are the branches of the tree. The branches of the tree are composed of the four clusters obtained by considering the partition that immediately precedes the absolute maximum level (cut-off) of inter-cluster dissimilarities (see Figure 1). This partition was chosen. The associated distance of the generalised affinity coefficient separates the estimated ARIMA models that best fit the carbapenem resistance values of *Klebsiella pneumoniae* across several EU countries into four distinct groups at the cut-off. For this partition the Silhouette coefficient (0.75) reaches a high value. This value suggest a relatively strong and well-defined clustering structure.

**Figure 2:**
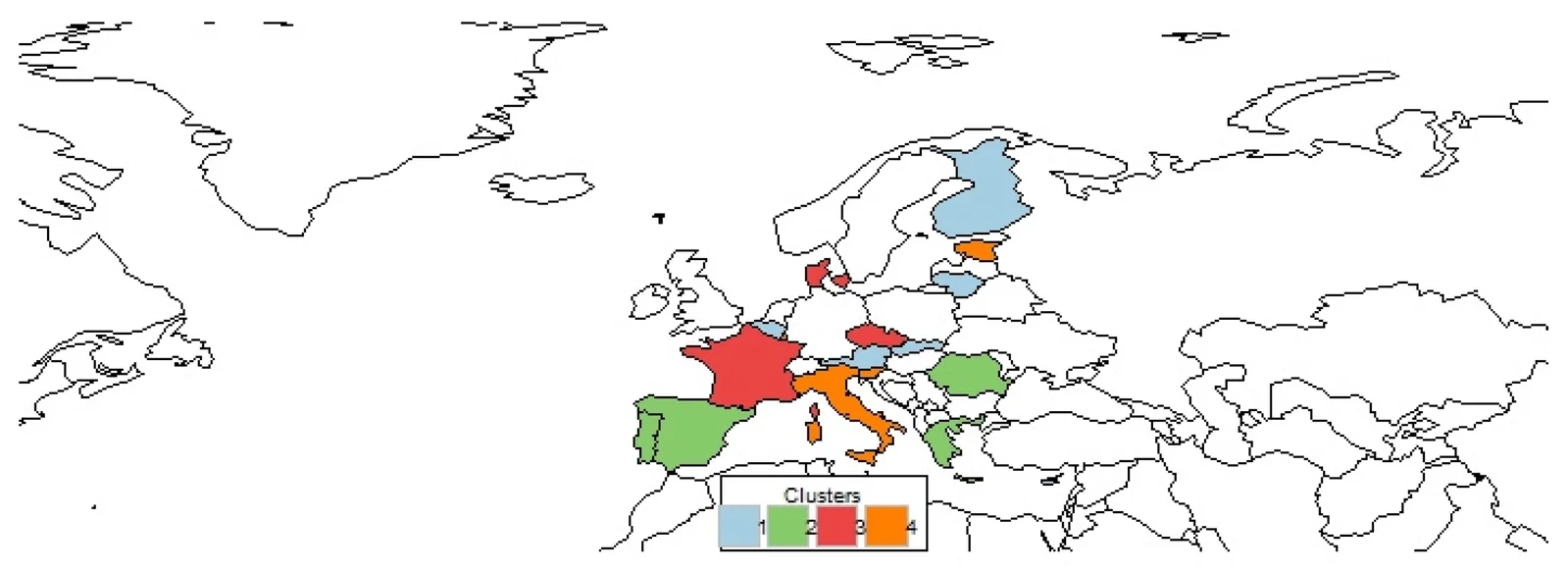
Map representing four clusters from cluster analysis in *K. pneumoniae*|carbapenem resistance context.

The map displaying the countries coloured according to their respective clusters is presented in Figure 2.

**Figure 3:**
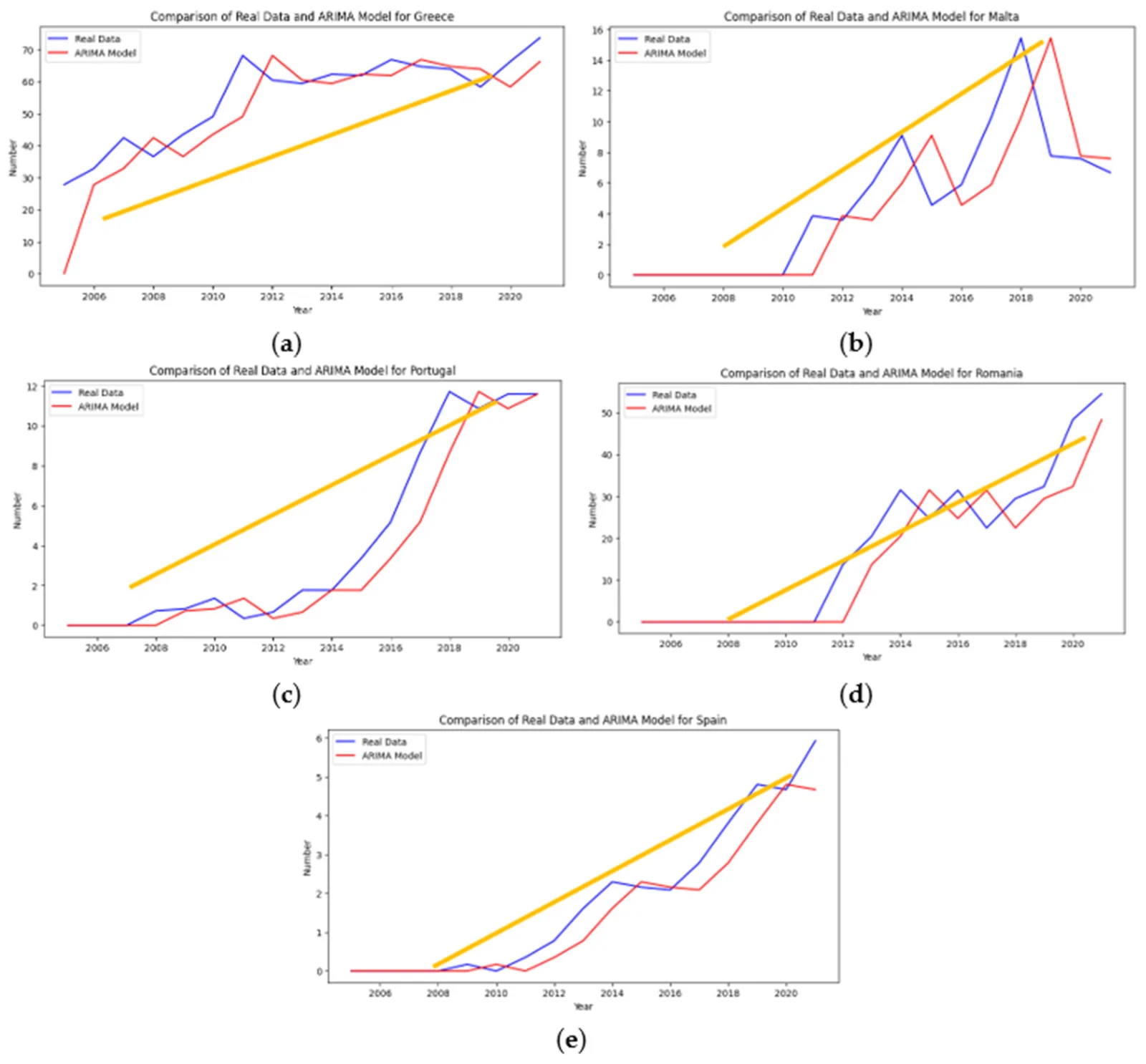
Real data and fit ARIMA model for *K. pneumoniae*|carbapenem resistance between 2005 to 2021 in (**a**) Greece; (**b**) Malta; (**c**) Portugal; (**d**) Romania and (**e**) Spain. The yellow line represents the trend over the study period.

The following figure (Figure 3) presents the estimated ARIMA models in comparison with the observed data for each EU country included in cluster II. In the figure, the x-axis represents time in years, while the y-axis represents resistance values. Note that the scale of resistance values varies from country to country, as the magnitude of these values differs depending on the country.

**Figure 4:**
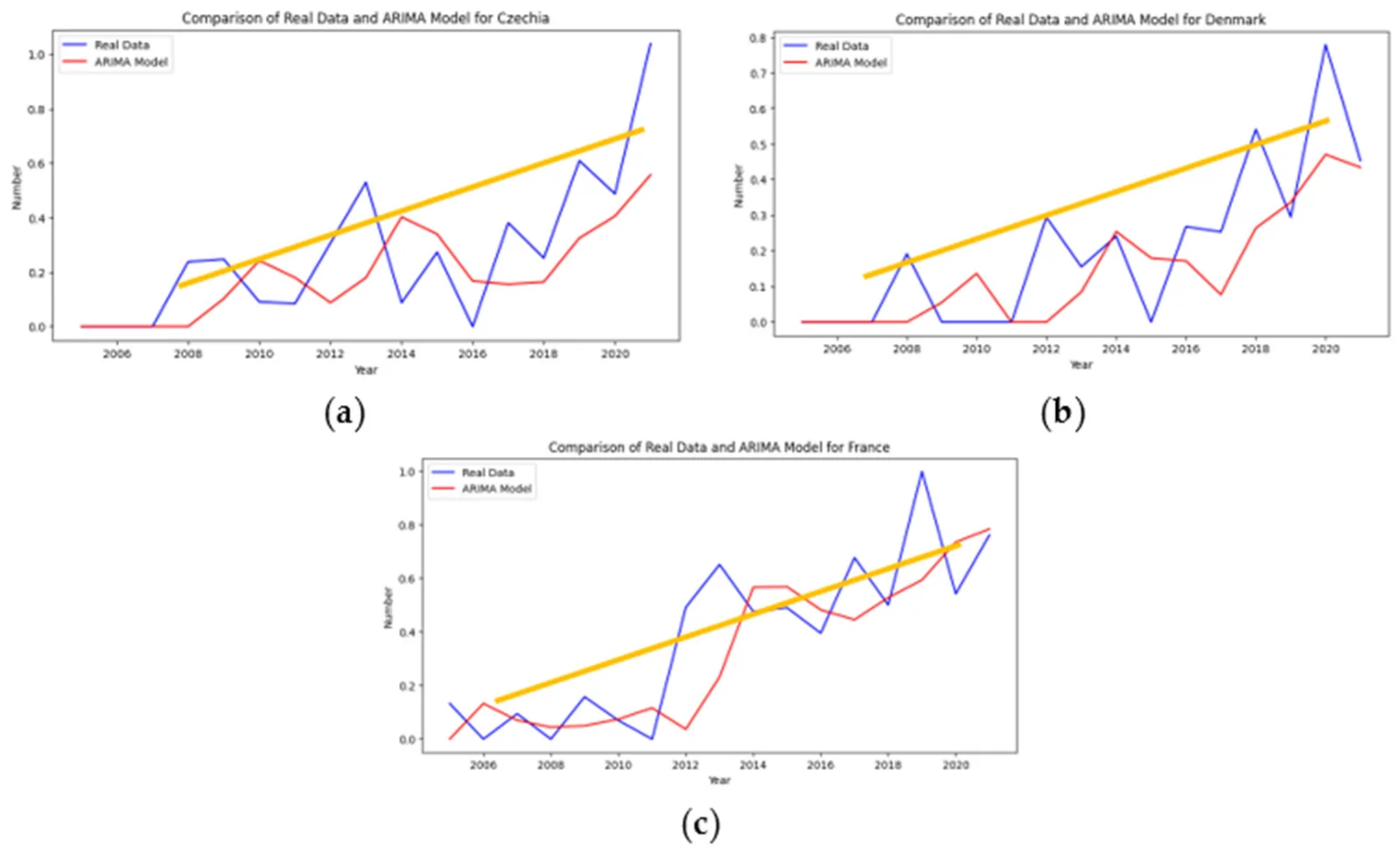
Real data and fit ARIMA model for *K. pneumoniae* |carbapenem resistance between 2005 to 2021 in (**a**) Czechia, (**b**) Denmark and (**c**) France. The yellow line represents the trend over the study period.

Figure 4 presents the estimated ARIMA models in comparison with the observed data for each EU country included in cluster III.

Although resistance values show an increasing trend over time in all clusters, this increase is more pronounced in clusters II and III. The data were standardised because the magnitudes of the resistance were different among countries, and the slopes of the lines between 2012 and 2020 were calculated for clusters II and III. For clusters I and IV, this was not possible due to the irregularity of the curves. The slopes were higher in the countries belonging to cluster II. Specifically, the slopes for cluster II (Figure 3) are m = 0.29, m = 1.5, m = 0.06, m = 0.11, and m = 0.26 for Portugal, Spain, Greece, Malta, and Romania, respectively, whereas for cluster III (Figure 4), the slopes are m = 0.02, m = 0.08 and m = 0.001 for France, Czechia and Denmark, respectively. It is also worth noting that within cluster II, Portugal, Romania and Spain show a steeper increase, with Spain showing the most pronounced growth. It is imperative that these countries strengthen their capacity to address this issue.

## 3. Discussion

The new generalised affinity coefficient between ARIMA models was successfully applied to AMR data in several EU countries, effectively addressing the issue of missing data and identifying the group of countries where intervention by health authorities is most urgently needed. In many cases, the AMR data did not have the same length, meaning there were missing data points. One way to address this issue is to treat each time series as a single object, rather than considering the series as multiple data points. This involves using the fitted model for each time series instead of the series itself. Therefore, in the specific case of the time-series data of *K. pneumoniae* resistance to carbapenems in various countries, which vary in length, it was more appropriate to apply agglomerative HCA based on the generalised affinity coefficient. Applying this generalised affinity coefficient also allowed the formation of groups of countries based on the evolution of their health indicators over time, enabling a clear assessment of model reliability and resistance dynamics in diverse national contexts and facilitating the identification of comparable patterns in public health outcomes. Treating each time series as a single object is advantageous as it enables the analysis of series with different lengths, facilitates the identification of temporal trends in in *Klebsiella pneumoniae* resistance to carbapenems, and supports the discovery of meaningful patterns within the data. This new coefficient allows the temporal structure of the data to be considered, while being less sensitive to extreme values than the Euclidean distance. Two countries with similar temporal dynamics but different magnitudes may not be considered similar when using the Euclidean distance. In contrast, the associated distance of the generalised affinity coefficient isolates the structural behaviour of the ARIMA model. This approach provides a clearer image of how disease burdens change over time, rather than focusing only on their magnitude. Furthermore, HCA based on the generalised affinity distance yielded a higher Silhouette coefficient (0.75) for the best partition than HCA using the Piccolo distance [20,21], whose best partition yielded a Silhouette coefficient of 0.64. This finding indicates a more cohesive and better-separated clustering structure when the generalised-affinity-based distance is employed.

Furthermore, the use of these tools provides an overview and contributes to a better understanding of overall behaviour, facilitating decision-making, particularly in this specific field.

Based on the results obtained using the generalised affinity coefficient that accounts for temporal behaviour, cluster II, comprising Portugal, Spain, Romania, Malta and Greece, stands out due to its increasing resistance trend, making it imperative for these countries to strengthen their capacity to address this issue. The adoption of robust and coordinated policies may be crucial in mitigating the spread of carbapenem resistance and safeguarding public health systems. In the countries belonging to the other groups, it is also necessary to control the incidence of this type of resistance by implementing measures to prevent its spread, since all groups, despite their different resistance patterns, show an overall increasing trend.

The application of data analysis, for the first time, using HCA based on the generalised affinity coefficient allowed the evaluation of patterns among different regions and the identification of clusters in which *K. pneumoniae* resistance to carbapenems was increasing over time. Based on slope calculations, Spain (m = 1.5) shows the most pronounced increase in *K. pneumoniae* resistance to carbapenems, with a mean resistance value of 1.85%. This trend is particularly evident in cluster II, which includes Spain, highlighting a group of countries in which resistance increased over time and may therefore be relevant for continued epidemiological surveillance and for reinforcing dissemination prevention strategies. Although Greece had lower slopes, Greece, Italy and Romania had the highest values of resistance (see Table 1). Greece and Romania, as members of cluster II, also showed a sustained upward trend over time. These temporal patterns may be relevant for continued epidemiological surveillance and require further investigation to better understand the factors underlying the observed trends. The clustering analysis identified groups of countries with similar temporal patterns of antimicrobial resistance. In particular, some clusters were characterised by increasing resistance trends over the study period, indicating a concerning temporal pattern in the prevalence of resistance to carbapenems of *K. pneumoniae*. These findings highlight countries and groups of countries in which resistance increased over time; however, the identification of increasing resistance trends does not, by itself, establish the need for specific interventions or indicate which prevention and control strategies should be implemented. The observed temporal patterns may be influenced by several factors, including antimicrobial consumption, infection prevention and control practices, surveillance intensity, laboratory practices, healthcare-related factors, and other epidemiological determinants, which were not evaluated in the present study. Therefore, the identified clusters should be interpreted primarily as groups of countries with similar temporal resistance patterns rather than as evidence of specific public health interventions being required.

Further studies incorporating epidemiological, healthcare, antimicrobial consumption, and surveillance-related variables are needed to investigate the factors underlying the observed resistance trends and to determine their potential implications for public health policy.

## 4. Materials and Methods

As already stated in the Results Section, data on resistant bacteria/antimicrobial agent was obtained from the public website of European Centre for Disease Prevention and Control (ECDC) [26].

The dataset comprises the resistant isolate percentage values of *K. pneumoniae*|carbapenem pairs reported in several EU countries from 2005 to 2021. It should be noted that the recording of AMR data for some countries may have started after 2005. Thus, the time-series data have different lengths, and there is missing data.

The EU countries considered in this paper are Austria, Belgium, Cyprus, Czechia, Denmark, Estonia, Finland, France, Greece, Italy, Lithuania, Luxembourg, Malta, Portugal, Romania, Slovakia, Slovenia, Spain.

The mean and standard deviation for *K. pneumoniae* resistance to carbapenems were calculated. These summary statistics enabled the characterisation of the obtained clusters.

Agglomerative HCA was employed to explore patterns and relationships among various countries in context of AMR, particularly for pair *K. pneumoniae*|carbapenem. These data are time series of different lengths. Then, linear interpolation was used to replace the missing values. Linear interpolation was performed using the *interpolate* function in Python version 3.13.7. To perform cluster analysis, the ARIMA models that best represented the behaviour of resistant isolate percentage values over time for the *K. pneumoniae*|carbapenem pair in several countries were obtained. Model selection was made by the optimisation of the Akaike Information Criterion (AIC) and Bayesian Information Criterion (BIC), which penalise excessive complexity to ensure model parsimony. The model was then validated by the Ljung–Box test to ensure there was no autocorrelation that indicated white noise behaviour. The residual autocorrelation was assessed using the Ljung–Box test automatically reported in the *summary ()* of the ARIMA model estimated using *statsmodels*. In this output, the test is presented as Ljung–Box (L1), indicating that the test was performed up to the first lag. The Ljung–Box Q statistic is calculated based on the residual autocorrelations and jointly assesses whether they are significantly different from zero. The null hypothesis states that there is no residual autocorrelation up to the specified lag. Therefore, *p*-values greater than 0.05 indicate no statistically significant evidence of residual autocorrelation, suggesting that the model adequately captures the temporal dependence present in the data.

The process to obtain the ARIMA models that best represent the data and the steps to evaluate those models were carried out in Python using the *pmdarima* package. Wrapping different statistical and machine learning libraries, *pmdarima* allows generalising ARIMA models into a single class. The *autoARIMA* function automates model selection by conducting a grid search over various parameter values for p and q, selecting the model that minimises AIC or BIC. For parameter d, the function uses a test of stationarity and seasonality for seasonal models. This efficient process significantly reduces time spent on model selection by automating the search for optimal parameters [27].

To measure the similarity among countries in the context of AMR, in the case of the *K. pneumoniae*|carbapenem pair, a new similarity measure, namely, the generalised affinity coefficient, between ARIMA models was applied. This new similarity measure is less sensitive to extreme values compared to the Euclidean distance. Agglomerative HCA, based on the new generalised affinity coefficient, was applied to the estimated ARIMA models representing the temporal trends of resistance over time for the *K. pneumoniae*|carbapenem pair in several European countries.

**Figure 5:**
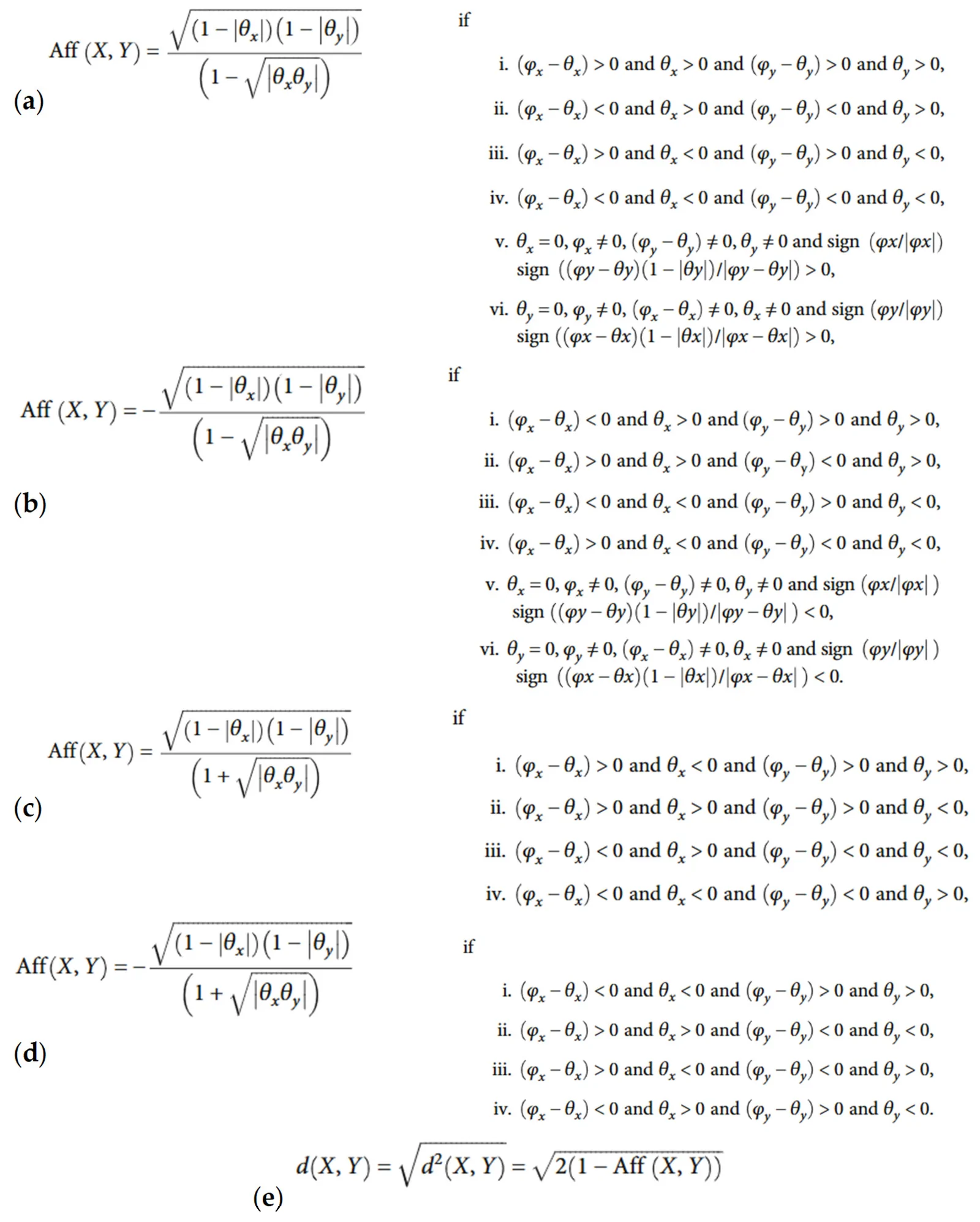
(**a**–**d**) New generalised affinity coefficient for AR (1), MA (1), ARMA (1,1) and (**e**) new associated distance of generalised affinity coefficient.

The new generalised affinity coefficient proposed for AR (1), MA (1), ARMA (1,1) (conditions (a) to (d) on Figure 5) and the new associated distance of the generalised affinity coefficient ((e) on Figure 5) were determined using the programming language R and is given in previous work by the authors [13]:

Agglomerative HCA was performed with average linkage criterion using *hclust* function of *stats* package of R. The average linkage method, for example, produces more balanced hierarchies and uses more information [28,29].

Agglomerative HCA results may be visualised through graphical representations called dendrograms or cluster trees, where the distance between clusters is reflected by the height of the dendrogram [29].

To select the clusters, it should be emphasised that the advantage of using HCA derives from the possibility of analysing the hierarchy of partitions, taking into consideration the branches of the dendrogram and the way in which they are generated, rather than focusing exclusively on individual partitions. Therefore, the clusters highlighted through visual inspection of the dendrogram are considered first and interpreted in terms of the various partitions that emerge throughout the clustering process. The successive partitions show how the pairs of observations within the partition under consideration are progressively merged. For the partition under consideration, whether it corresponds to the “best absolute partition” of the hierarchy, namely, a partition that immediately precedes the absolute maximum in inter-cluster dissimilarities, was assessed, as indicated by the height of the dendrogram [16]. Therefore, the partition that immediately precedes the absolute maximum level (cut-off) in inter-cluster dissimilarities was chosen [16]. To evaluate the chosen partition, the Silhouette coefficient was calculated [29,30,31].

The Silhouette coefficient has shown good results with real data [32]. Therefore, throughout this work, the value of this coefficient was considered.

The Silhouette coefficient evaluates cluster quality by analysing the cohesion within clusters and the separation between clusters. It quantifies the similarity of each data unit to other members of its own cluster compared with its similarity to data units in the other clusters. Values close to 1 indicate that, in each partition, data units are more similar to the members of their own cluster than to those belonging to other clusters, suggesting that they are well-assigned. Conversely, values close to −1 indicate that data units are more similar to members of other clusters than to those of their own cluster, suggesting an incorrect cluster assignment. Values close to 0 indicate that a data unit may lie between clusters and could belong to a different cluster [33]. Therefore, the coefficient is close to 1 when clusters are dense and well-separated.

**Figure 6:**
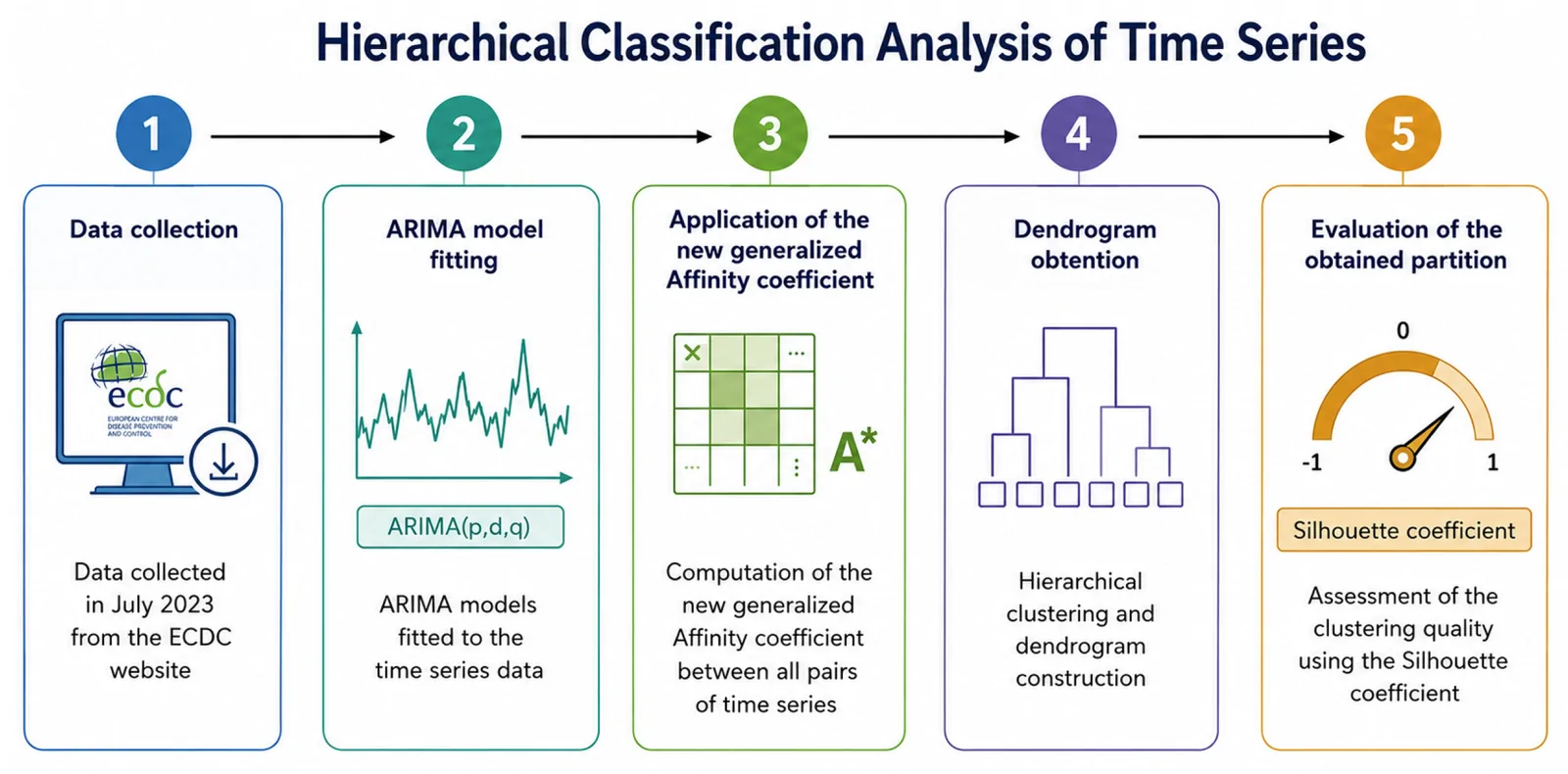
Hierarchical classification analysis of time series. Methodological workflow comprises five sequential steps: (1) data collection from the European Centre for Disease Prevention and Control (ECDC) website in July 2023; (2) fitting of ARIMA models to time-series data; (3) application of new generalised affinity coefficient to quantify similarity between pairs of time series; (4) hierarchical clustering and dendrogram construction based on resulting affinity measures; (5) evaluation of the obtained partition using Silhouette coefficient to assess clustering quality. A*—represents the affinity coefficient. Figure generated with AI.

Figure 6 summarises the methodological pathway.

For better visualisation of potential geographical patterns, a world map with the cluster membership between countries was made with the package *rworldmap*.

## 5. Limitations of This Study

Several limitations of this study should be acknowledged. First, the biological and epidemiological validation and interpretation of the identified clusters may have been influenced by differences in surveillance systems, laboratory practices, testing methodologies, and other countries or setting specific factors that could affect the comparability of antimicrobial resistance data.

In addition, information on the number of isolates available at each time point was not accessible. Consequently, the mean resistance values used in the analysis may have been affected by differences in sample size over time, with estimates based on smaller numbers of isolates potentially being less representative and more susceptible to random variation. Nevertheless, a minimum of 30 isolates at each timepoint was assured.

Although the limited length of the annual time series did not allow a reliable cross-validation procedure, the *autoarima* function provides a systematic and objective approach to model selection by evaluating multiple combinations of model parameters and identifying the optimal specification according to information criteria such as AIC or BIC. Therefore, despite this limitation, the selected model can be considered the most appropriate ARIMA model for the available data.

## 6. Conclusions

The application of data analysis using HCA based on the generalised affinity coefficient between ARIMA models, allowed the evaluation of the temporal trends among countries, allowing the identification of clusters that may need intervention. The results obtained were particularly higher for the countries in cluster II, with Spain standing out. Based on slope calculations, Spain (m = 1.5) showed the most pronounced increase in *K. pneumoniae* resistance to carbapenems, with a mean resistance value of 1.85%. Although Greece exhibited lower slopes, it had the highest resistance values, as did Italy and Romania. Greece and Romania, as members of cluster II, showed a sustained upward trend over time, also placing them among the countries requiring enhanced monitoring.

The results obtained in the present work demonstrate the need for additional research to clarify the factors contributing to the observed trends. Further studies incorporating epidemiological, healthcare, antimicrobial consumption, and surveillance-related variables, among others, are needed to investigate the factors underlying the observed resistance trends and to determine their potential implications for public health policy.

## Figures and Tables

**Table 1:** Mean and standard deviation of *K. pneumoniae* resistance to carbapenems in several countries in the EU.

Country	Mean (%)	SD (%) ^1^
Austria	0.59	0.46
Belgium	0.93	0.63
Cyprus	12.6	7.1
Czechia	0.29	0.28
Denmark	0.22	0.23
Estonia	0.32	0.72
Finland	0.11	0.18
France	0.38	0.30
Greece	55.2	13.73
Italy	22.04	13.07
Lithuania	0.63	1.11
Luxembourg	0.50	0.70
Malta	5.04	4.47
Portugal	4.69	4.75
Romania	25.74	16.42
Slovakia	4.18	3.43
Slovenia	0.34	0.40
Spain	1.85	1.96

^1^ SD, standard deviation.

## Data Availability

The data of resistant bacteria/antimicrobial agent were obtained from the public ECDC website, “Surveillance Atlas of Infectious Diseases”, https://Atlas.Ecdc.Europa.Eu/Public/Index.Aspx (accessed on 23 July 2023).

## Abbreviations

The following abbreviations are used in this manuscript:

AMR	Antimicrobial resistance
ARIMA	Autoregressive Integrated Moving Average
ECDC	European Centre for Disease Prevention and Control
HCA	Hierarchical cluster analysis

## Appendix A

The ARIMA models that best fit the resistant isolate percentage values data for *K. pneumoniae*|carbapenem are presented in Table A1.

The ARIMA models were selected as the best-fitting models based on the Akaike Information Criterion (AIC) and Bayesian Information Criterion (BIC). Model selection was performed by minimising these criteria, which balance goodness of fit against model complexity by penalising the inclusion of unnecessary parameters. Consequently, the selected models provide the simplest representation of the data while maintaining a high level of explanatory power [27].

**Table A1:** The ARIMA models that best fit the resistant isolate percentage values data for *K. pneumoniae*|carbapenems.

Country	Model	Phi/Theta ^1^
Austria	ARIMA (0,1,1)	0/−0.3843
Belgium	ARIMA (0,1,1)	0/−0.6110
Cyprus	ARIMA (1,0,0)	0.9474/0
Czechia	ARIMA (1,1,0)	−0.6264/0
Denmark	ARIMA (1,1,0)	−0.7862/0
Estonia	ARIMA (0,0,1)	0/0.4842
Finland	ARIMA (1,0,0)	0.6291/0
France	ARIMA (1,1,0)	−0.5290/0
Greece	ARIMA (0,1,0)	0/0
Italy	ARIMA (0,1,1)	0/0.6047
Lithuania	ARIMA (1,0,0)	0.5876/0
Luxembourg	ARIMA (0,0,1)	0/0.7199
Malta	ARIMA (0,1,0)	0/0
Portugal	ARIMA (0,1,0)	0/0
Romania	ARIMA (0,1,0)	0/0
Slovakia	ARIMA (0,1,1)	0/−0.5073
Slovenia	ARIMA (0,0,1)	0/0.9215
Spain	ARIMA (0,1,0)	0/0

^1^ Phi, autoregressive parameter estimate; theta, moving average parameter estimate.

Table A2 presents the Ljung–Box test statistics and their corresponding *p*-values for the models that best fit the resistant isolate percentage data for *K. pneumoniae* resistant to carbapenems.

**Table A2:** The ARIMA models that best fit the resistant isolate percentage values data for *K. pneumoniae*|carbapenems with Ljung–Box statistics and respective *p*-values.

Country	Model	Ljung–Box/*p*-Value
Austria	ARIMA (0,1,1)	0.85/0.36
Belgium	ARIMA (0,1,1)	0.03/0.86
Cyprus	ARIMA (1,0,0)	0.26/0.61
Czechia	ARIMA (1,1,0)	0.20/0.66
Denmark	ARIMA (1,1,0)	0.0/0.98
Estonia	ARIMA (0,0,1)	0.04/0.84
Finland	ARIMA (1,0,0)	0.10/0.75
France	ARIMA (1,1,0)	0.54/0.46
Greece	ARIMA (0,1,0)	0.22/0.64
Italy	ARIMA (0,1,1)	0.38/0.54
Lithuania	ARIMA (1,0,0)	0.06/0.80
Luxembourg	ARIMA (0,0,1)	0.27/0.60
Malta	ARIMA (0,1,0)	0.0/0.98
Portugal	ARIMA (0,1,0)	0.06/0.80
Romania	ARIMA (0,1,0)	1.77/0.18
Slovakia	ARIMA (0,1,1)	2.66/0.10
Slovenia	ARIMA (0,0,1)	0.02/0.89
Spain	ARIMA (0,1,0)	0.02/0.89
